# In Vitro Screening of Ecotoxic and Cytotoxic Activities of *Ailanthus altissima* Leaf Extract against Target and Non-Target Plant and Animal Cells

**DOI:** 10.3390/ijms25115653

**Published:** 2024-05-22

**Authors:** Maria Denisa Cocîrlea, Natalia Simionescu, Anca Roxana Petrovici, Mihaela Silion, Barbara Biondi, Luana Lastella, Simona Oancea

**Affiliations:** 1 Department of Agricultural Sciences and Food Engineering, “Lucian Blaga” University of Sibiu, 7–9 Dr. Ion Ratiu Street, 550024 Sibiu, Romania; denisa.cocirlea@ulbsibiu.ro; 2 Centre of Advanced Research in Bionanoconjugates and Biopolymers, “Petru Poni” Institute of Macromolecular Chemistry, 41A Aleea Grigore Ghica-Voda, 700487 Iasi, Romania; natalia.simionescu@icmpp.ro (N.S.); petrovici.anca@icmpp.ro (A.R.P.); 3 Physics of Polymers and Polymeric Materials Department, “Petru Poni” Institute of Macromolecular Chemistry, 41A Grigore Ghica Voda Alley, 700487 Iasi, Romania; silion.mihaela@icmpp.ro; 4 Institute of Biomolecular Chemistry, Padova Unit, Consiglio Nazionale delle Ricerche (CNR), Via Marzolo 1, 35131 Padova, Italy; barbara.biondi@unipd.it; 5 Department of Chemistry, University of Padova, Via Marzolo 1, 35131 Padova, Italy; luana.lastella@unipd.it

**Keywords:** *Ailanthus altissima*, ecotoxicity, cytotoxicity, germination test, shrimp lethality assay, liposome, cancer cells

## Abstract

*Ailanthus altissima*, an invasive plant species, exhibits pharmacological properties, but also some allergic effects on humans. This study aimed to evaluate the potential toxicity of *A. altissima* leaves, using a complex approach towards different organisms. The ecotoxic impact of a crude extract was investigated on seeds germination and brine shrimp lethality. Cytotoxicity was studied in vitro using non-target (haemolysis, liposomal model, fibroblast), and target (cancer cells) assays. Leaf extract at 1000 µg/mL significantly inhibited wheat and tomato germination, while no significant effects were found on parsley germination. A slight stimulatory effect on wheat and tomato germination was found at 125 µg/mL. In a brine shrimp-test, the extract showed a low toxicity at 24 h post-exposure (LC_50_ = 951.04 ± 28.26 μg/mL), the toxic effects increasing with the exposure time and extract concentration. Leaf extract caused low hematotoxicity. The extract was biocompatible with human gingival fibroblasts. No anti-proliferative effect was found within the concentration range of 10–500 µg/mL on malignant melanoma (MeWo) and hepatocellular carcinoma (HepG2). In a liposomal model-test, the extract proved to possess low capability to alter the eukaryotic cell-mimicking membranes within the tested concentration range. Given the low to moderate toxicity on tested organisms/cells, the *A. altissima* autumn leaves may find useful applications.

## 1. Introduction

*Ailanthus altissima* (Mill.) Swingle (Simaroubaceae), also called Tree of Heaven or China-sumac, is a perennial tree native to Asia, which has been introduced into new areas as an ornamental plant. In the course of time, *A. altissima* has become invasive producing a strong impact on local plant communities and soil characteristics [1]. This species has been included on the List of Invasive Alien Plants by the European and Mediterranean Plant Protection Organization (EPPO) [2] since 2004. The spreading of *A. altissima* is difficult to control despite the fact that several chemical and mechanical removal strategies have been proposed [3].

On the other hand, a great number of scientific papers have reported the bioactivities of *A. altissima* confirming several important biological properties of different parts of the plant, e.g., the antimicrobial properties of its leaves [4,5], the neuroprotective effects of its bark [6], the anti-inflammatory properties of its seeds, branches and leaves [7], and the DNA protective effects of its flowers, stem bark and leaves [8]. With regard to its composition, the root barks of *A. altissima* contain alkaloids, triterpenoids, lignans and coumarins, the stem barks contain mainly terpenoids (e.g., ailanthone) [9,10,11], while the fruits and seeds are rich in terpenoids [12], quassinoid glycosides [13], steroids [14,15] and phenolics [16]. The leaves of *A. altissima* are known for their high content of polyphenols, molecules with strong antioxidant properties, followed by lower amounts of other compounds, such as alkaloids, sesquiterpene and triterpenoids (ailanthone), proteins, carbohydrates, minerals, etc. [17,18], making leaves of such species excellent candidates for diverse applications (antimicrobials, biopesticides) [19]. However, extracts from such invasive plant species have been less investigated for their level of impact on the environment or human health, generally because natural products are mostly perceived as safe. Yet, it is known that numerous plants synthesize highly toxic metabolites [20].

The particular interest in the pesticidal effects of natural extracts makes imperative the evaluation of their eco-safety to non-target organisms because such products will be intentionally liberated into the environment. Ecotoxicity is usually conducted towards crop plants and organisms from aquatic ecosystems. Ecotoxicological studies have revealed some effects of *A. altissima* aqueous extract on wheat [21] or other plants’ germination [22,23,24,25,26], and on the mortality of the *Daphnia magna* crustacean [27]. Cytotoxicity on mammalian cells is usually investigated either on cell cultures exposed to natural products when referring to their safety to non-target organisms, or screening tests on tumor cells when searching for potential bioactive agents. Among cytotoxicological assessments, the in vitro hemolysis assay has been applied as a simple and cheap method to study the disruption of erythrocyte membranes induced by a chemical compound or a natural extract [28]. A similar test but on model lipid membranes has also been studied for medicinal and food applications [29,30]. The cytotoxicity of *A. altissima* against several tumor cells, such as HepG2 [31,32], HeLa [31,33], 786-O [31], A549 [31,34,35], Jurkat [36], R-HepG2 [37], MCF-7 [34,38], Hep3B [37], MDA-MB-231 [34,39], LAPC4, A375, B16 [40], and SGC-790 [41], has been reported.

Our present study aims to give complete information on the potential toxic effects of *A. altissima* leaves using a complex approach to different organisms (target/non-target organisms), serving as a key step for future safety considerations of these extracts for diverse applications. The ecotoxic effects of an ethanolic extract of *A. altissima* leaves on plants (wheat, tomato, parsley) were investigated using the seed germination inhibition test, and on crustaceans using the crustacean *Artemia salina* lethality test. The cytotoxic effects were studied using different assays, non-target (hemolysis, membrane leakage assay from lipid vesicle by fluorescent spectroscopy, MTS assay on human gingival fibroblast HGF), and target (MTS assay on malignant melanoma and hepatocellular carcinoma). To our knowledge, screening the toxicity of *A. altissima* leaf extracts using the brine shrimp, hemolysis, membrane leakage, malignant melanoma cells and HGF assays has not been reported so far.

## 2. Results and Discussion

### 2.1. Characterization of Phenolic Compounds of A. altissima Leaf Extract by HPLC-DAD

In the present work, we investigated the ecotoxic and cytotoxic effects of an *A. altissima* leaf hydroethanolic extract, which has been characterized by a total content of 6026.31 ± 4.89 mg gallic acid equivalents/100 g DW (phenolics), 575.50 ± 0.10 mg catechin equivalents/100 g DW (tannins), 55.60 ± 0.11 mg β-carotene/100 g DW (carotenoids) and a total antioxidant capacity as measured by FRAP assay of 5043.59 ± 48.11 mg ascorbic acid equivalents/100 g DW.

**Figure 1:**
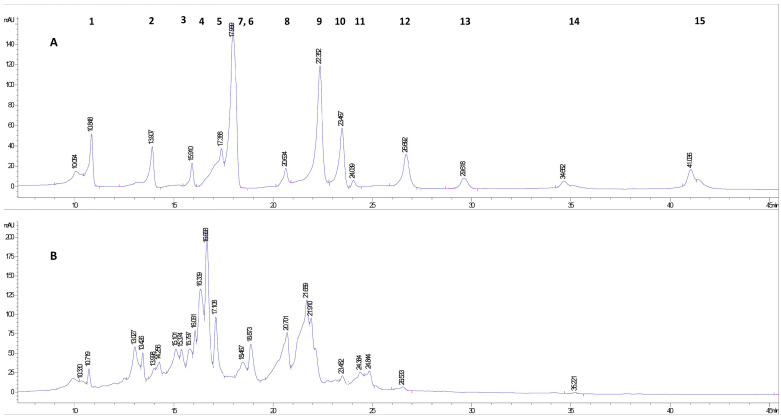
The comparative HPLC-DAD chromatograms of polyphenolic compounds; (**A**)—Standards: 1—Gallic acid, 2—Protocatechuic acid, 3—Catechin, 4—Vanillic acid, 5—Epicatechin, 6—Caffeic acid, 7—Syringic acid, 8—Rutin, 9—Ferulic acid, 10—*p*-Coumaric acid, 11—Hesperidin, 12—Rosmarinic acid, 13—Salicylic acid, 14—Quercetin, 15—Kaempferol; (**B**)—crude leaf extract.

For the identification of polyphenols in *A. altissima* leaf extract, stock solutions of 15 polyphenols were prepared with a concentration of 0.5 mg/mL. Using the separation method developed by our research group for HPLC analysis, all 15 polyphenols compounds were analyzed one by one. Retention times (RT) are listed in Table 1. After their analysis, all the 15 polyphenols were mixed in equal proportions and the mixture was analyzed using the same HPLC separation method. Following the separation, as noticed in the HPLC chromatogram (Figure 1), a slight shift in RTs occurred (Table 1) due to the inter-molecular interactions between all 15 compounds in the solution. At the same time, because the RTs were very close for four of the compounds, their peaks overlapped two by two (see Table 1). Even if these compounds’ peaks overlapped in the mixture spectrogram, in the spectrogram obtained for the leaf extract the separation was evident for epicatechin and vanillic acid.

Following the performance of the HPLC analysis, nine polyphenolic compounds were identified in the *A. altissima* hydroethanolic extract obtained from dried autumn leaves, of which five were phenolic acids (gallic acid, protocatechuic acid, vanillic acid, *p*-coumaric acid, rosmarinic acid) and four were flavonoids (catechin, rutin, hesperidin, quercetin). All the polyphenolic compounds identified in this study were previously reported by other authors in the leaves of this species [8,42,43].

### 2.2. Ecotoxicity of A. altissima Leaf Extract

#### 2.2.1. Inhibitory Effects of *A. altissima* Leaf Extract on Germination of Seeds Used in Agricultural Crops

Ecotoxicity assessments using plant assays are simple approaches providing results suitable for statistical analysis and in connection with those obtained from testing on animal models or even human cells, being used either to identify environmental contaminants or to preliminarily validate drugs and pesticides [44,45]. Wheat and tomato are plant species approved for the ecotoxicity testing of products by the Organization for Economic Cooperation and Development (OECD) and the Food and Drug Administration (FDA) [46].

##### Wheat Caryopsis Germination Test

Wheat, an important global agricultural crop [47,48], represents one of the most commonly used species for the toxicity assessment of chemical compounds, natural extracts, and nanoparticles [45,49,50].

The results on wheat germination and growth records in the presence of *A. altissima* ethanolic extract at a concentration range 125–1000 μg/mL are presented in Table 2, comparatively to those for the control.

Most germination parameters excepting the shoot length showed higher mean values in the presence of the lowest extract concentration (125 μg/mL) among all investigated samples. Most germination parameters excepting the germinative energy and shoot length decreased in the presence of the highest extract concentration (1000 μg/mL).

**Figure 2:**
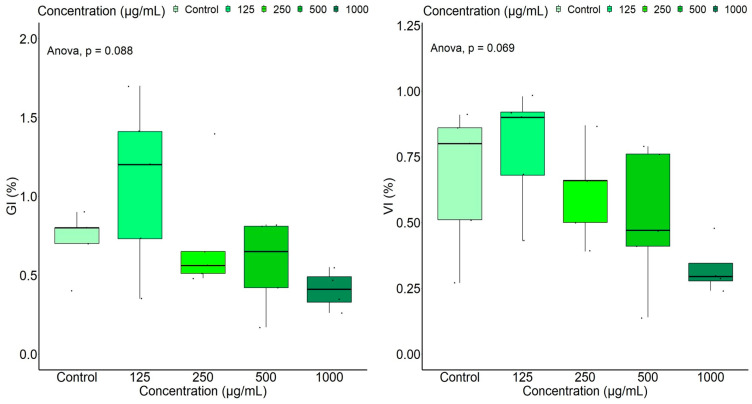
Boxplot representation of the measured/calculated wheat germination indices, according to extract concentration.

By conducting an ANOVA test, marginally significant differences were observed for GI (%) (*p* = 0.088) and VI (%) (*p* = 0.069) (Figure 2) in relation to the extract concentration. According to the Tukey test, the vigor index varied significantly between groups with an added extract at 125 µg/mL and 1000 µg/mL, respectively (*p* = 0.0490), while the germination index varied marginally (*p* = 0.0625). Although the ANOVA and Kruskal–Wallis tests did not show statistically significant differences for the other germination parameters related to extract concentrations and to the control (*p* > 0.1), the most pronounced inhibitory effect of leaf extract on wheat was found at the highest investigated concentration, 1000 μg/mL, while the sample at the lowest concentration (125 μg/mL) actually produced an opposite effect—a slight stimulation of wheat caryopses for all measured indices compared to those of the control and samples with higher concentrations.

**Figure 3:**
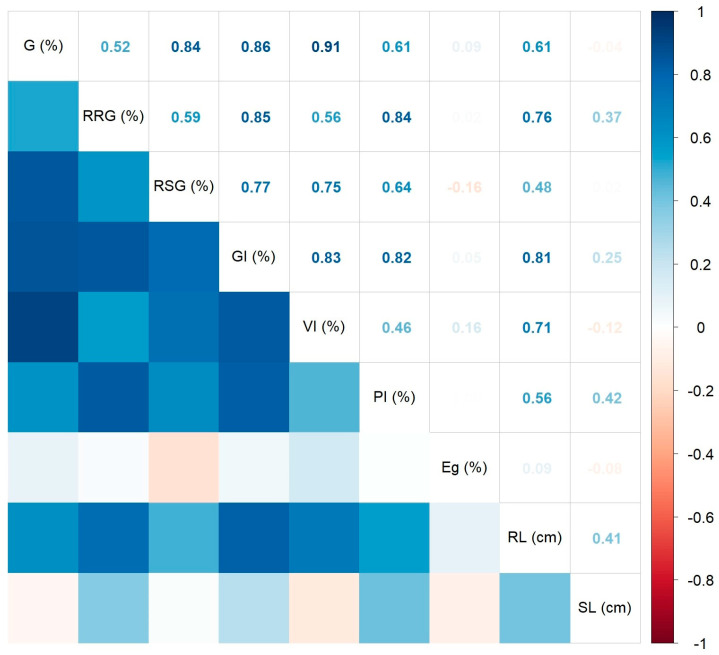
Correlation plot of the Spearman’s correlation coefficients between the measured/calculated wheat germination indices.

The Spearman’s correlation coefficients between various measured/calculated germination indices are presented in Figure 3 by using a correlogram. As noticed, all significant coefficients at *p* < 0.05 showed positive values. The strongest correlations were found between the germination rate and vigor index (R^2^ = 0.91, *p* < 0.01). The weakest significant correlation was identified between the root length and shoot length (R^2^ = 0.41, *p* = 0.0484). The germination energy showed no statistically significant correlation with the other variables, while the shoot length indicated only one significant positive correlation with the influence index on the aerial part (R^2^ = 0.42, *p* = 0.0437), and a marginally significant one with the relative root growth (R^2^ = 0.37, *p* = 0.0778).

Ahmad et al. (2020) [21] observed that an aqueous extract of *A. altissima* leaves may adversely affect the wheat root length and caryopsis germination. The study of Novak et al. (2021) [51] on *A. altissima* but using aqueous extracts from roots and an aqueous solution of the pure compound ailanthone, reported very weak inhibitory activity on the wheat germination and root length growth compared to the effect it had on other tested plant seeds (pigweed *Amaranthus retroflexus* L. and red bristlegrass *Setaria pumila* L.), at lower concentrations. The authors found that the least significant effect was shown by ailanthone, indicating that an inhibitory effect occurs only in the presence of other root allelochemicals (synergism). According to other published papers, *A. altissima* root or leaves revealed either an inhibitory effect on *Medicago sativa* [24,25], *Daucus carota* [22], *Sinapis alba* and *Brassica napus* [23], *Lactuca* sp. [26], or a stimulating effect on *Raphanus sativus* L. and *Setaria pumila* L. [51,52]. The inhibitory effects may be further studied for their potential application as bioherbicides [52].

##### Tomato Seed Germination Test

The tomato species (*Lycopersicum esculentum*), belonging to the Solanaceae family, has been used in order to determine the phytotoxic activity of various plant extracts [53,54,55] or antibiotics [56], being considered an appropriate species due to its sensitivity to toxic substances, availability, and good germinating rate [46,57].

Considering the results on the wheat caryopses germination test, two concentrations (125 and 1000 μg/mL, respectively) of the ethanolic leaf extracts of *A. altissima* were investigated for their potential inhibitory activity on tomato germination. The results are presented in Table 3, comparatively to those for the control.

Most germination parameters excepting the germination rate, relative seed germination, and vigor index, showed the highest mean values in the presence of the lowest extract concentration (125 μg/mL) among all investigated samples. Most germination parameters excepting the germinative energy decreased in the presence of the highest extract concentration (1000 μg/mL).

**Figure 4:**
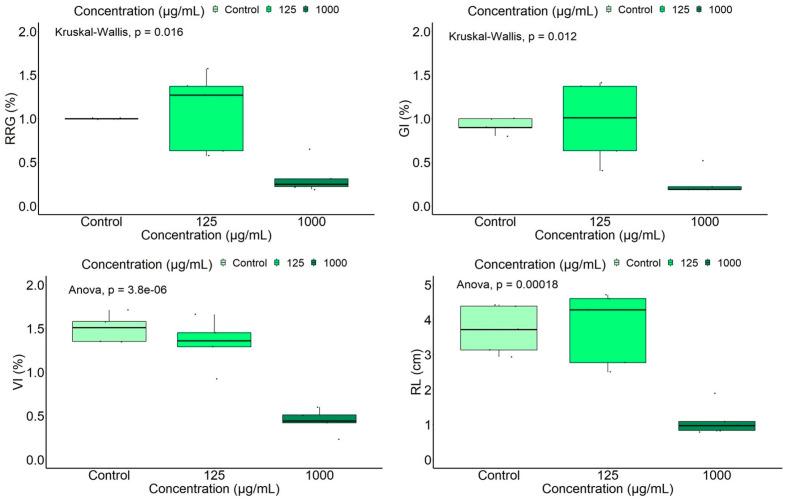
Boxplot representation of the measured /calculated tomato germination indices, according to the extract concentration.

A boxplot representation of four germination/ growth indices (RRG, GI, VI, RL) which revealed significant differences in relation to extract concentrations and the control is shown in Figure 4.

Data analysis by the ANOVA test and its non-parametric variant, the Kruskal–Wallis test, showed significant differences between mean values of germination indices as a function of extract concentration, indicating a decrease in the investigated indices with the increase in extract concentration. A high statistically significant inhibitory effect was registered for the group with added extract at 1000 μg/mL. No statistically significant differences were found between germination indices of the control and those in the group with added leaf extract at the lowest concentration, 125 μg/mL. Similarly, the Dunn’s test indicated highly statistically significant differences (*p* < 0.05) for the indices RRG and GI between the groups with added leaf extract at different concentrations, and between the group with the highest extract concentration and the control. The Tukey’s test showed statistically significant higher VI and RL values (*p* < 0.001) in the control and the group with 125 μg/mL added extract than those in the group at the highest tested concentration, 1000 μg/mL. Although the Kruskal–Wallis/ ANOVA analysis showed no statistically significant differences between groups with added extract at different concentrations for the indices Eg (*p* = 0.1346), RSG (*p* = 0.6138), PI (*p* = 0.5312), G (*p* = 0. 613), and SL (*p* = 0.559), slightly lower values were observed at 1000 μg/mL and higher values at 125 μg/mL compared to the control. As an exception, the mean germination energy was slightly higher in the group with the highest extract concentration than in the control, and the mean germination rate showed close values at all concentrations. Our results showed that the root length was more affected by the extract concentration, compared to the aerial part length.

**Figure 5:**
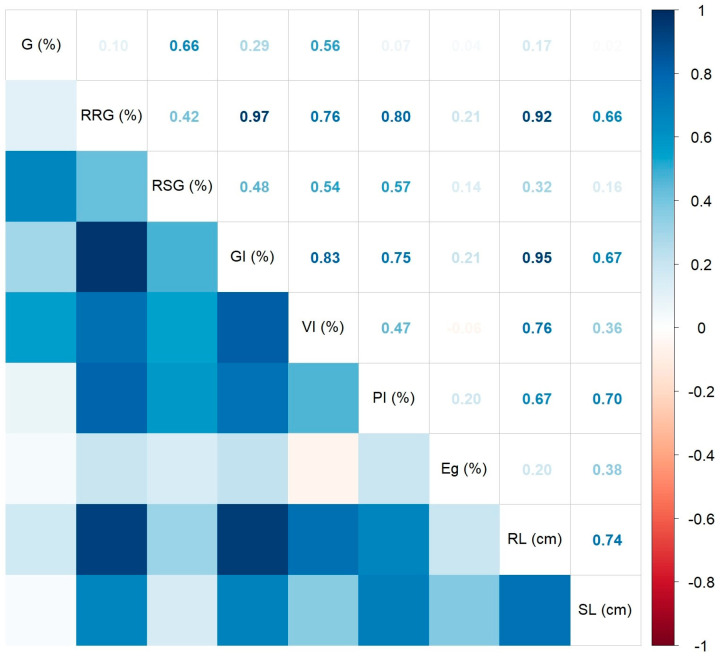
Correlation plot of the Spearman’s correlation coefficients between the measured/calculated tomato seed germination indices.

All Spearman’s correlations in the *L. esculentum* seed germination test were positive and significant (*p* < 0.05), as shown in the correlogram of Figure 5. The strongest statistically significant correlations were found between the RRG and GI (R^2^ = 0.97, *p* < 0.01), and between the GI and RL (R^2^ = 0.95, *p* < 0.01). The weakest significant correlation was identified between the RSG and VI (R^2^ = 0.54, *p* = 0.0370). All correlations of the Eg parameter were not statistically significant.

In the study of Heisey and Heisey (2003) [58], the compound ailanthone isolated from *A. altissima*, which was applied to the tomatoes in the field, did not significantly influence the production of tomatoes or their biomass.

###### Parsley Seed Germination Test

Parsley (*Petroselinum crispum* (Mill.) var. *crispum*) represents an important herb for the food industry and gastronomy [59,60], known as a fast-growing species [61]. Most reported studies have focused on improving its germination characteristics because of the low germination rate of these seeds [62,63,64].

Similarly to the experimental runs on the tomato seed germination test, two concentrations (125 and 1000 μg/mL, respectively) of ethanolic leaf extract of *A. altissima* were investigated for their potential inhibitory activity on parsley seed germination. The results are presented in Table 4, comparatively to those for the control.

All the investigated germination parameters in samples with added leaf extracts were lower than those in the control sample.

**Figure 6:**
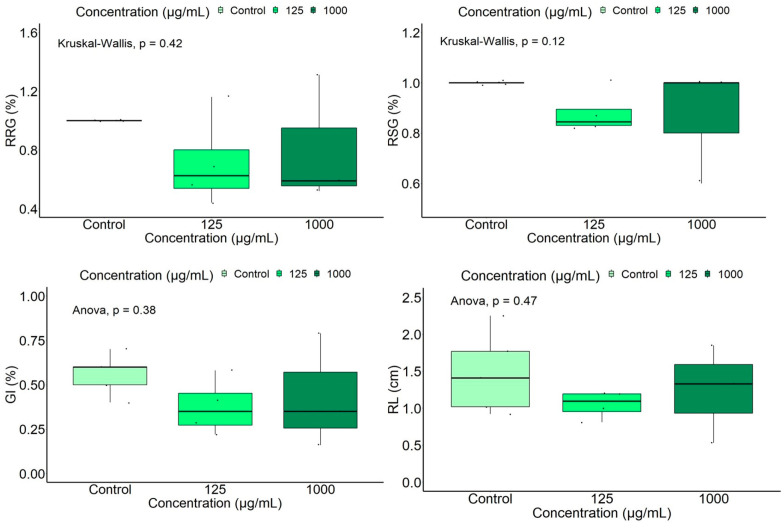
Boxplot representation of the measured/calculated parsley germination indices, according to the extract concentration.

A boxplot representation of four germination/growth indices (RRG, RSG, GI, RL) in relation to extract concentrations and the control is shown in Figure 6.

As shown in Figure 6, no statistically significant differences (*p* ˃ 0.05) were found between values of the investigated germination indices between control and groups with added leaf extracts, and within groups with different concentrations of extracts, by using ANOVA and Kruskal–Wallis tests. Despite the lower values of germination indices in the presence of the plant extract, compared to those in the control, no significant inhibitory effect of ethanolic extract of *A. altissima* leaves was identified on parsley seed germination.

**Figure 7:**
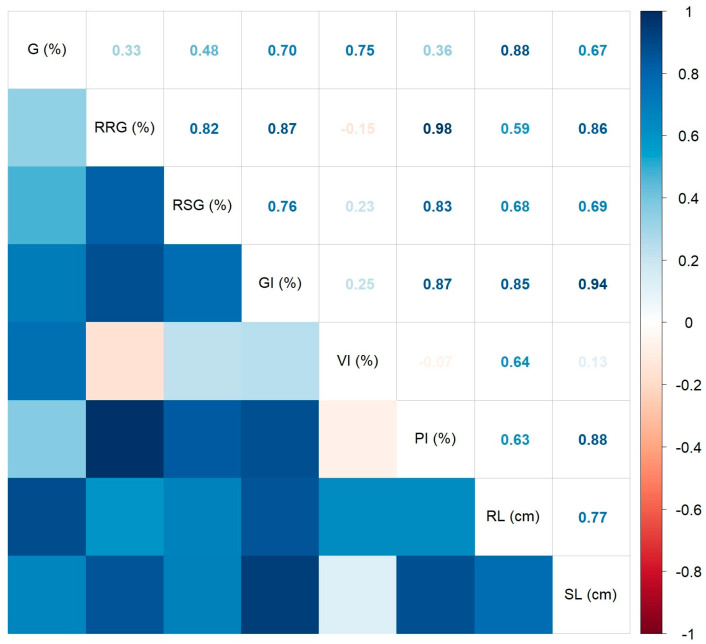
Correlation plot of the Spearman’s correlation coefficients between the measured/calculated parsley seed germination indices.

All Spearman’s correlations in the parsley seed germination test were significant and positive (Figure 7). The strongest significant correlations were found between the RRG and PI (R^2^ = 0.98, *p* < 0.01), and between the GI and SL (R^2^ = 0.94, *p* < 0.01). The weakest significant correlation was found between the RRG and RL (R^2^ = 0.59, *p* = 0.0414).

The effect of *A. altissima* extracts on parsley seed germination has not been reported so far. Therefore, our results could not be compared to other similar ones.

Regarding the effects of different concentrations of *A. altissima* leaf extract on the germination of seeds used in agricultural crops (wheat, tomato, parsley), our results indicate an inhibitory effect of the extract at the highest tested concentration (1000 μg/mL) on wheat germination (only marginally significant results, 43–51% for the GI and VI) and tomato germination (statistically significant, 67–72% for the RRG, GI, VI, RL) compared to the control, while no statistically significant effects were found using the parsley germination test. The statistically significant decrease in germination indices (RRG, GI, VI, RL) of the tomato with increased extract concentration might be due to the fact that tomatoes behave like bioindicators and are much more sensitive to the presence of allelochemicals than wheat, as shown by Vidotto et al. (2013) [65]. At the highest investigated extract concentration, the development of seedlings of the three crop plants was also altered, showing a more negative impact on the growth of the root than that of the aerial part. This observation has been also reported by Asgharipour and Armin (2010) [66], in their study on an aqueous extract of *Sorghum halepens*, which inhibited to a greater extent the root growth of *Ocimum basilicum* than the shoot length. Using the tomato and wheat germination test, we observed a slight stimulatory effect of 7–8% with respect to the germinative energy in the presence of *A. altissima* leaf extract.

#### 2.2.2. Inhibitory Effects of *A. altissima* Leaf Extract on *A. salina* Hatching

The ecotoxicity of products towards aquatic organisms is usually tested against crustaceans, e.g., *D. magna*, *Daphnia pulex*, *Scapholeberis kingi* or *A. salina* [20], being regulated for the risk assessment of chemicals and materials by international organizations such as the American Society for Testing and Materials (ASTM), the OECD and EU [67,68,69], or for acute toxicity evaluation by the US-EPA [70]. Originally described by Meyer et al. (1982) [71], the brine shrimp larvae (BSL) test becomes over time an easy-to-assess standard technique with promising results in detecting the toxicity and anti-proliferative activity of plant extracts [72,73,74,75] to be applied in the pharmaceutical industry [76].

**Figure 8:**
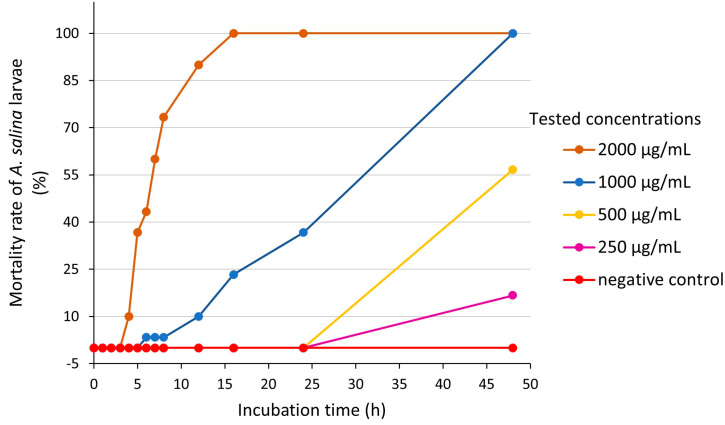
Evolution of the cumulative mortality rate of *A. salina* larvae incubated with different concentrations of *A. altissima* leaf extract.

To further investigate the ecotoxicity of *A. altissima* leaves, we performed the assay on brine shrimp. The results regarding the evolution of the mortality rate of *A. salina* larvae incubated with different concentrations (250–2000 μg/mL) of *A. altissima* leaf extract are presented in Figure 8.

Throughout an incubation period of 48 h, no *A. salina* individual died in the negative control group, so that no correction by Abbott’s formula was required [77]. The extract concentration of 2000 μg/mL determined the shortest time to onset toxic effects, after 3 h of exposure, followed by the concentration of 1000 μg/mL, at which the larvicidal effects appeared after 5 h. Lower concentrations of extract (250 and 500 μg/mL) produced mortality only after 24 h of exposure.

The values of the median lethal concentration (LC_50_, μg/mL) of the leaf extracts on BSL using the Log Concentration by Probit analysis are presented in Table 5.

**Figure 9:**
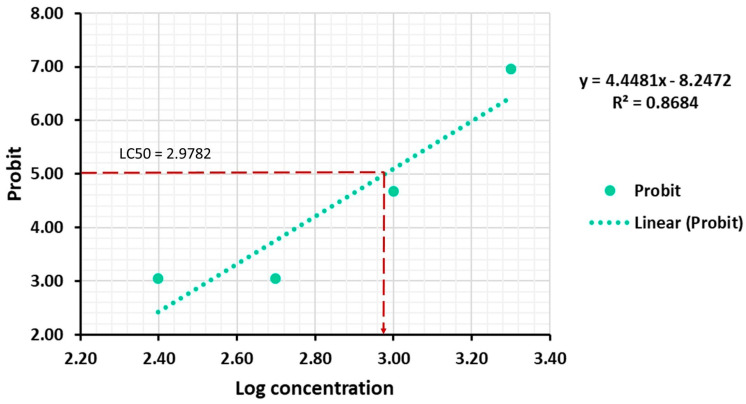
Probit graph for LC_50_ of *A. altissima* leaf extract against *A. salina*, after 24 h of exposure.

We found a strong positive correlation between the mortality rate and the extract concentration (R^2^ = 0.8684, *p* = 0.0681) after 24 h of exposure. The median lethal concentration (LC_50_) of *A. altissima* leaf extract, corresponding to a Log concentration of 2.9782, was 951.04 ± 28.26 μg/mL (24 h of exposure). As shown in Figure 9, the equation of the linear regression of the mortality rate expressed in probability units (Probit) against the logarithm of the concentration was Y= 4.4481X − 8.2472.

The lower the LC_50_ value is, the more toxic the compound/extract is [77]. The toxicity of the ethanolic extract of *A. altissima* leaves on *A. salina* at 24 h was low, according to Clarkson’s toxicity criterion: extracts with LC_50_ ˃ 1 mg/mL, non-toxic; LC_50_ = 0.5–1 mg/mL, low toxicity; LC_50_ = 0.1–0.5 mg/mL, medium toxicity; and LC_50_ = 0–0.1 mg/mL, highly toxic [80].

**Figure 10:**
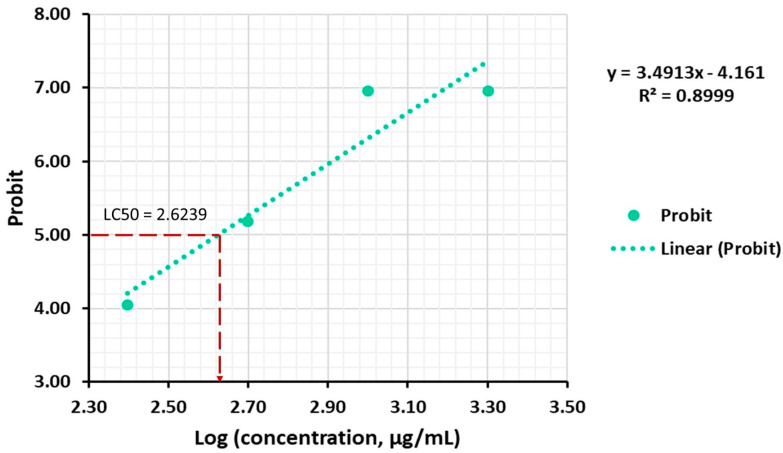
Probit graph for LC_50_ value of *A. altissima* leaf extract against *A. salina*, after 48 h of exposure.

We also found a strong positive correlation between the mortality rate and the extract concentration (R^2^ = 0.8999, *p* = 0.0514) after 48 h of exposure. The median lethal concentration (LC_50_) of *A. altissima* leaf extract, corresponding to a Log concentration of 2.6239, was 420.65 μg/mL (48 h), corresponding to a medium toxicity according to Clarkson’s toxicity criterion [80]. As shown in Figure 10, the equation of the linear regression of the mortality rate expressed in probability units (Probit) against the logarithm of the concentration was Y = 3.4913X − 4.161.

To our knowledge, no study regarding the *A. altissima* toxicity towards *A. salina* has been published so far. The toxicity of fresh *A. altissima* leaves litter extracts has been tested towards another aquatic invertebrate, *D. magna*, showing a median effective concentration (EC_50_) of 10.1 g/L of air-dried leaf, at 96 h [27]. In our study, the sensitivity of *A. salina* to *A. altissima* extract increased with time, the highest one being registered after 48 h, similar to other studies on natural extracts but from different species, e.g., from marine organisms [81]. The mortality of *A. salina* individuals was positively correlated with the concentration of the extract to which they were exposed, similar to that reported in the study of Krishnaraju et al. (2016) [82] on aqueous extracts of some Indian medicinal plants, including the species *Ailanthus excelsa.*

### 2.3. Cytotoxicity of A. altissima Leaf Extract

The study of the interaction between lipid membranes and bioactive molecules or natural extracts allows one either to identify their potential inhibitory effect on microbial and cancer cells, or to evaluate their cytotoxic effects [83]. Hereby, we investigated the interaction of leaf extracts with different cells (erythrocytes, fibroblasts, cancer cells) and with animal cell model membranes, considering it a key initial step to evaluate extract toxicity.

#### 2.3.1. Hemolytic Activity

**Figure 11:**
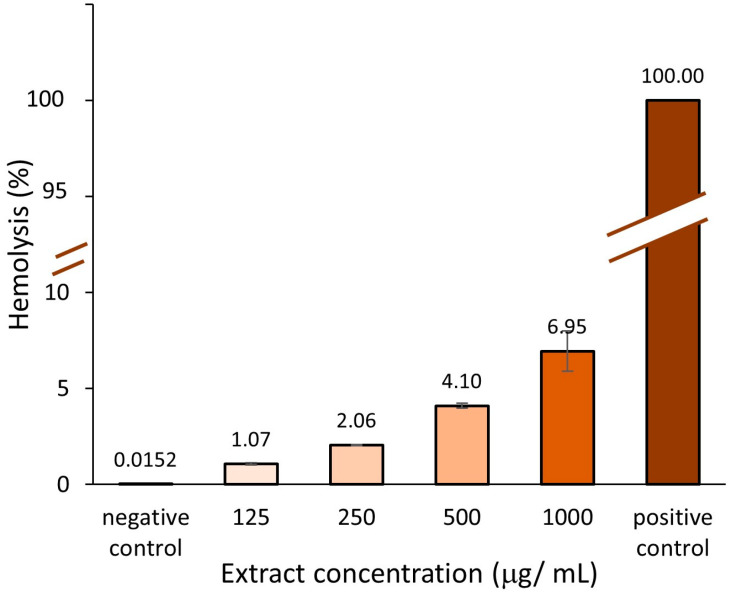
Hemolytic activity of leaf extract at different concentrations.

The effect of different concentrations of *A. altissima* leaf extract ranging from 125 to 1000 μg/mL, on sheep erythrocyte membrane, was investigated using the erythrocyte viability assay, frequently applied to test the safety of drugs and medicinal nanoparticles [84,85,86]. The results are shown in Figure 11.

The hemolytic activity, HC_50_, calculated as the value at which 50% of erythrocytes are lysed in the presence of the extract [87], was determined. Based on a regression calculation, according to the equation Y = 0.0067X + 0.414 from plotting hemolysis versus concentration, the HC_50_ value was 7400.9 µg/mL (equivalent to 7.4%, *w*/*v*).

Increasing the concentration of the leaf extract will increase hemolysis. None of the investigated concentrations of leaf extract produced a significant hemolytic activity. The highest hemolysis (6.95 ± 1.04%) occurred in the presence of 1000 μg/mL.

There are no guidelines on the hemolytic properties of natural extracts, but the hemolytic properties of materials used in medical devices, such as nanomaterials, are regulated according to the ASTM based on the percentage of human erythrocyte lysis: <5% (no hemolysis), 5–10% (low hemolysis) ˃10% (marked hemolysis) [88]. Translating these to *A. altissima* leaf extract, our results indicate a non-hemolytic limit concentration of 500 μg/mL. No hemolytic studies have been reported for *A. altissima*. In the study of Silva et al. (2023) [89], the synthetic pure coumarin D, a chemical compound found in *A. altissima* bark being of interest for its antifungal properties, showed a low hemolytic effect (<5%), while several analogs of it determined even less hemolysis. However, the authors indicated that the results cannot exempt the extract from showing toxicity towards other types of cells in the human body. More than being low hemolytic, some natural compounds in particular polyphenols displaying antioxidant properties, showed protective effects on induced erythrocyte hemolysis [84,90]. Caffeic acid and tannins, which are polyphenolic compounds frequently found in plants including *A. altissima* leaves [5,91], have revealed a strong capacity to inhibit induced hemolysis [92,93].

#### 2.3.2. In Vitro Biocompatibility and Cytotoxicity of Leaf Extract at Different Concentrations

Cell viability experiments are important tests in toxicity studies offering information on the cellular response to a toxicant. MTS—a tetrazolium salt-based colorimetric assay—was used in the present study to evaluate the proliferation activity of cells after 24 h incubation.

**Figure 12:**
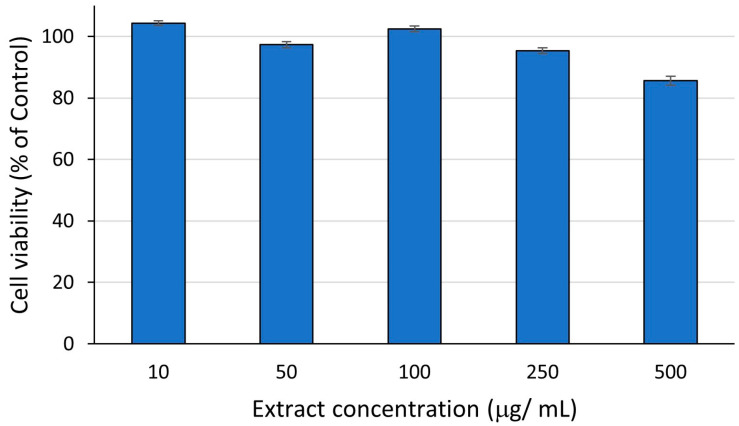
Biocompatibility of *A. altissima* leaf extracts on human fibroblasts (HGF) after 24 h; data were represented as means ± standard error of the mean.

The results regarding the biocompatibility test of *A. altissima* leaf extracts in the concentration range 10–500 μg/ mL performed on human gingival fibroblast (HGF) are presented in Figure 12.

In this study, *A. altissima* leaf extract was biocompatible up to 500 µg/mL. The level of fibroblast viability decreased by increasing the concentration of *A. altissima* extract. The extract at 10 μg/ mL showed the highest percentage of HGF cell viability at 24 h, 104%. The lowest cell viability value (86%) was registered in the presence of 500 μg/ mL extract.

To our knowledge, this is the first report on the influence of *Ailanthus altissima* leaf extract on the viability of HGF cells.

**Figure 13:**
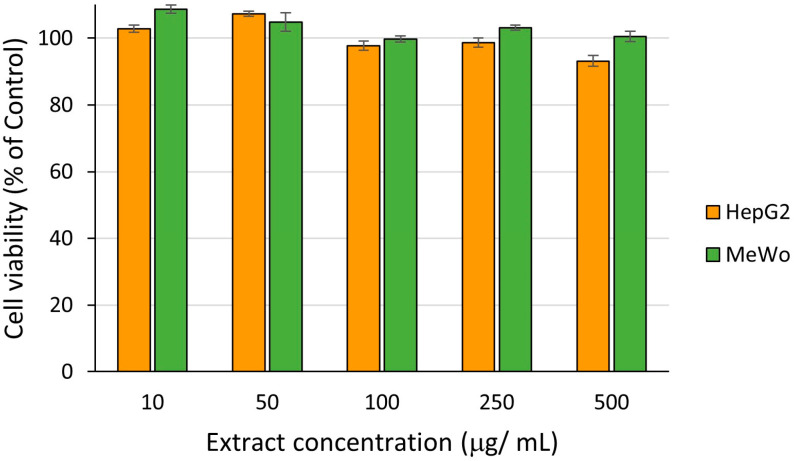
Cytotoxicity of *A. altissima* leaf extracts on malignant melanoma (MeWo) and hepatocellular carcinoma (HepG2) cells after 24 h; data were represented as means ± standard error of the mean.

Our further cytotoxic study was extended to target organisms and performed on hepatocellular carcinoma (HepG2) and malignant melanoma (MeWo) cells. The results are presented in Figure 13.

The extract had no cytotoxic effect on HepG2 and MeWo cells’ viability over 24 h incubation, indicating no anti-proliferative properties at tested concentrations and on the investigated cell lines. We calculated the half-maximal inhibitory concentration value, IC_50_, of the extract from the MTS assay for HepG2 and MeWo cells by using the regression equation. IC_50_ for HepG2 cells was 2441.63 µg/mL, while for MeWo cells it was 5124.07 µg/mL.

Mohamed et al. (2021) [32] also observed a weak cytotoxic effect of the methanolic fraction of a crude extract from *A. altissima* leaves after 72 h of exposure on HepG2 cells, all the other tested fractions (EtOAc and *n*-BuOH fractions) exhibiting lower effects than those of doxorubicin, a standard drug [32]. According to the classification proposed by the U.S. National Cancer Institute (NCI) and Geran protocol, which the authors employed in their paper, a half-maximal inhibitory concentration (IC_50_) between 200 µg/mL and 500 µg/mL was associated with a weak citotoxicity [32]. In the study of Gao et al. (2022) [94], a new steroid with the molecular formula C_24_H_34_O_4_ isolated from leaves of *A. altissima* demonstrated an important antiproliferative effect on HepG2 cells, with an IC_50_ = 4.03 μM, more effective than the antiproliferative drug sorafenib. To our knowledge, no studies reported any investigation of *A. altissima* leaf extract on MeWo cells, but published research showed that quercetin, a polyphenolic compound of flavonoid class, inhibits the signaling and expression of the c-Met receptor in wild-type melanoma MeWo [95].

#### 2.3.3. Model Membrane-Modifying Properties of *A. altissima* Leaf Extract

A simple and relatively stable lipid membrane model of artificial liposomes can be used instead of living cells for studying the interaction of natural extracts with lipid vesicles of various sizes e.g., small unilamellar vesicle (SUV, 20–50 nm in diameter), large unilamellar vesicle (LUV, 100–500 nm in diameter), giant unilamellar vesicle (GUV 10–100 µm in diameter), multilamellar vesicle (MLV, >500 nm in diameter) and multivesicular vesicle (MVV, >500 nm in diameter) [29,30].

**Figure 14:**
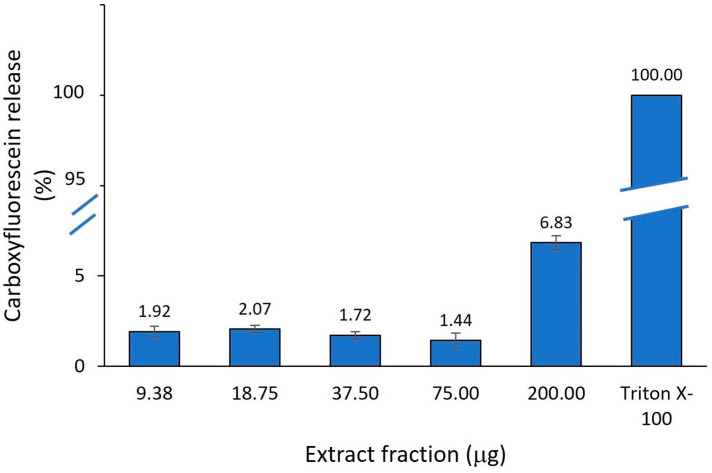
Leaf extract-induced leakage of carboxifluorescein (CF) trapped within phosphatidylcholine/ cholesterol SUVs at 20 min for different [extract]/ [lipid] fractions.

In order to corroborate our findings presented above, the permeability of lipid membranes prepared of 1,2-dioleyl-sn-glycero-3-phosphocholine and cholesterol (7/3) was investigated in the presence of different concentrations (125–1000 μg/mL) of *A. altissima* leaf extract by measuring the induced release of a fluorescent dye (carboxyfluorescein, CF) from small unilamellar liposomes. These liposomes represent a valuable model for eukaryotic cell membranes. The phospholipid concentration was kept constant (0.06 mM) and increasing [extract]/[lipid] molar ratios were obtained by adding aliquots of extract at concentrations between 125 and 1000 μg/mL. The results are presented in Figure 14.

Under the explored conditions, the *A. altissima* leaf extract did not exhibit any capability to alter or disrupt liposomes within the tested concentration range, even at the highest tested fraction (200 μg) when 6.83% of CF was released. The obtained results indicate a low toxicity of the extract on the investigated liposomal model, a eukaryotic cell-mimicking membrane. No other published papers studying the effect of *A. altissima* leaf extract on liposome leakage have been identified so far. However, changes in liposome permeability using calcein leakage assay have been recently reported for testing the toxicity of pesticides [96].

One reason why the membrane of liposomes deteriorates is the lipid oxidation [97]. However, the presence of large amounts of polyphenols in the natural extract is not sufficient to explain their possible protective effects on liposomal membranes, according to the observations made by Rodrigues et al. (2016) [98]. Among various phenolic compounds, the leaves of *A. altissima* contain gallic acid [22,99,100], which has a good capacity to protect membranes from oxidative stress [97].

## 3. Materials and Methods

### 3.1. Plant Material and Extract Preparation

Leaflets of *A. altissima* were collected in the autumn from Vâlcea county, Romania (45°6′34″ (N), 24°22′42″ (E)). The voucher specimen of this plant was deposited at the Herbarium (No. HFS 23 1053, Faculty of Sciences) of the “Lucian Blaga” University of Sibiu. The collected leaves were subjected to hot-air drying with forced air circulation (UFE 400, Memmert, Schwabach, Germany) at 50 °C such as to reach a final moisture content of 5–6% as measured using a moisture analyzer (Mac 210/NP Radwag, Radom, Poland). Dried leaves were ground using a knife mill (Grindomix GM 200, Retsch, Haan, Germany), sieved through a standard sieve (pore size 700 µm) and stored at 4 °C until analysis.

Ethanolic crude extracts of dried leaves were prepared by soaking the powdered plant sample into 70% aqueous ethanol solution at a sample/solvent ratio of 1/10 (*w*/*v*), for 6 h at r.t. in darkness. The mixture was centrifuged at 8000 rpm (Universal 320, Hettich, Berlin, Germany) for 10 min at 4 °C. The obtained supernatant was further concentrated using a centrifugal vacuum concentrator (RVC 2-18 CD plus, Christ, Munich, Germany) such as to obtain a soft extract (moisture < 15%).

### 3.2. Characterization of A. altissima Leaf Extract

The extract was subjected to chemical analysis of polyphenols, such as total phenolics using the Folin–Ciocalteu method [101], total condensed tannins by the vanillin assay [102], and carotenoids [103]. Antioxidant capacity was determined as well, using the Ferric Reducing Antioxidant Power (FRAP) assay described by Benzie and Strain [104].

HPLC-DAD analysis: the polyphenols’ separation and identification were achieved using an Agilent 1260 Infinity Series (Agilent Technologies, Santa Clara, CA, USA), equipped with a UV-Vis DAD detector and a binary pump. The compounds separation was performed using a Mediterranea Sea 18 column (4.6 mm × 150 mm, 5 μm particle size) (Teknokroma Analitica S.A., Barcelona, Spain) and a gradient elution was applied (Table 6) by using 0.1% (*v*/*v*) formic acid in water (mobile phase A), and acetonitrile (mobile phase B) (both purchased from Sigma-Aldrich Chemie GmbH, Taufkirchen, Germany), at 0.8 mL/min flow rate. 20 µL of each sample and standard was injected and the separation process was monitored by UV-VIS DAD detector at 280 nm. Fifteen polyphenolic compounds (gallic acid, protocatechuic acid, catechin, vanillic acid, epicatechin, caffeic acid, syringic acid, rutin, ferulic acid, *p*-coumaric acid, hesperidin, rosmarinic acid, salicylic acid, quercetin, kaempferol, all purchased from Sigma-Aldrich Chemie GmbH, Taufkirchen, Germany) were used as standards, from which 0.5 mg/mL stock solutions were prepared and injected both separately and as a mixture. The mixture was prepared by adding 100 μL of each stock solution, thoroughly homogenized and injected as such.

### 3.3. Determination of Ecotoxicity of A. altissima Leaf Extract

#### 3.3.1. Germination Bioassay of Wheat (*Triticum aestivum* L.) Caryopsis

*T. aestivum* organic seeds (Pronat, Romania) were subjected to sterilization according to the method described by Lindsey III et al. (2017) [105] and Dal Cortivo et al. (2017) [106] with slight modifications. A total of 100 seeds were covered with 500 μL sodium hypochlorite solution (50/50 *v*/*v*) for 5 min, after which they were rinsed seven times for 1 min with distilled water. A standard germination test was conducted in five replicates for each extract concentration and control. Ten wheat caryopses and 5 mL leaf extract of different concentrations (soft ethanolic extract re-suspended in distilled water such as to obtain 125, 250, 500 and 1000 μg/mL, respectively) were placed on a double layer of filter paper (79.7 g/m^2^, 0.165 mm thickness) in a glass Petri dish of 6 cm diameter, which was previously kept in a pre-heated oven at 140 °C for 30 min, in order to remove possible contaminating microorganisms. The control sample was prepared by using 5 mL distilled water. Incubation was conducted in an upright position of dishes at r.t. for 8 days, of which 6 days were monitored using the containers sealed with a tin foil, applied with the purpose of limiting the degradation of the active compounds of extract and the water evaporation, as suggested by Veisz et al. (1996) [107]. The measurements were made on the third day of germination when the number of sprouted caryopses was noticed, and on the eighth day when germinated seeds, root length and aerial length of seedlings were observed.

The following germination indices were evaluated: shoot length (SL, cm), root length (RL, cm), seed germination energy (Eg, %) [108], relative root growth (RRG, %) [109], influence index on the aerial part (PI, %) [110], vigor index (VI, %) and germination index (GI, %) [111], relative seed germination (RSG, %) and germination rate (G, %) [112]. The indices were calculated using the Equations (1)–(7):

(1) $$Eg\%=\frac{the\;number\;of\;germinated\;seeds\;in\;the\;third\;day}{the\;total\;number\;of\;seeds}$$

(2) $$G\%=\frac{the\;number\;of\;germinated\;seeds}{the\;total\;number\;of\;seeds}\times 100$$

(3) $$RRG\%=\frac{average\;root\;length\;for\;the\;sample}{average\;root\;length\;for\;the\;control}\times 100$$

(4) $$RSG\%=\frac{the\;number\;of\;germinated\;seeds\;in\;the\;sample}{the\;number\;of\;germinated\;seeds\;in\;the\;control}\times 100$$

(5) $$GI=\left(G\%\times RRG\%\right)\times 100$$

(6) $$VI=G\%\times \left(average\;root\;length+average\;stem\;length\right)$$

(7) $$PI=\frac{G\%\;sample-the\;average\;length\;of\;the\;aerial\;part\;for\;the\;sample}{G\%\;control-average\;aerial\;part\;length\;for\;control}\times 100$$

In case the groups contained fewer germinated caryopses than a minimum of three, they were excluded from the evaluation.

The root length of a seedling was determined by measuring each root strand from the root–shoot junction to the tip and calculated as an average value. The shoot length was obtained in the same way, but the measurement was taken from the culm’s base to the tip of a leaf.

#### 3.3.2. Germination Bioassay of Tomato (*Lycopersicum esculentum* Mill.) Caryopsis

This bioassay has been performed under similar experimental conditions as described for wheat caryopsis, with slight modifications: 3 mL leaf extract of two different concentrations (125 μg/mL and 1000 μg/mL, respectively) was added to 10 tomato seeds (Starsem, Universal, Romania) in the germination test. The measurements were made on the third and sixth day of germination.

#### 3.3.3. Germination Bioassay of Parsley (*Petroselinum crispum* (Mill.) var. Crispum) Seed

This bioassay has been performed under similar experimental conditions as described for tomato caryopsis. Parsley seeds from Starsen (Traditional) brand, produced by Agrosel SRL. (Romania), were used in the experiments. The measurements were made on the sixth day of germination, according to Ugolini et al. (2021) [113].

#### 3.3.4. Brine Shrimp (*Artemia salina*) Assay

The brine shrimp larvae (BSL) test was performed according to the method described by Apetroaei et al. (2018) [114]. For the experiment, commercially purchased *Artemia salina* eggs (Artemia Hobby; Manufacturer: DAJANA PET) were used. The eggs were hatched using a saline solution (38% *w*/*v*) according to McLaughlin et al. (1998) [74]. 500 mL saline solution and 5 g of *A. salina* eggs were added to a system consisting of two transparent plastic containers, the smaller one having a perforated base and being the one in which the eggs were placed and kept at 24 °C under light over 48 h. Larvae were transferred to identical transparent plastic containers from the hatching vessel using a pipette. Based on the description of McLaughlin et al. (1998) [74], 10 individuals/test container were counted. Although hatched larvae can survive 48 h without nutrient feeding [81], 50 μL of yeast suspension was added to each sample to limit the risk of larval death due to starvation [115]. To the vials containing brine shrimp, aliquots of leaf extracts obtained from *A. altissima* dried leaves were added at the following concentrations: 250, 500, 1000, and 2000 μg/mL. Experiments were carried out in triplicate per vial. The mortality rate was recorded up to 8 h on an hourly basis, and at 12, 16, 24, and 48 h, respectively. Larvae that showed no movement for at least 10 sec were considered dead, as suggested by Manfra et al. (2012) [116]. In the control group, the extract was replaced with distilled water, the experiments being conducted in three replicates.

The mortality rate was calculated according to Lam et al. (2020) [117], using the Equation (8):

(8) $$Mortality\;rate\;\left(\%\right)=\frac{number\;of\;the\;dead\;shrimps\;}{total\;number\;of\;experimental\;shrimps}\times 100$$

The median lethal concentration (LC_50_) was calculated according to Pohan et al. (2023) [118]. The transformation of mortality percentage into probability units (probits) was performed using a Finney’s table as described by Nigar et al. (2021) [119]. The transformation of 0% and 100% of mortality into probability units was carried out according to Randhawa (2009) [79].

### 3.4. Determination of Cytotoxicity of A. altissima Leaf Extract

#### 3.4.1. Hemolytic Activity Assay (Erythrocyte Viability)

The hemolytic activity of leaf extracts was determined according to the method described by Yamada et al. (1994) [120]. The experiments were conducted on sheep red blood cells (RBC) collected from farm animals of the Research and Development Institute for Montanology, Sibiu, Romania. The experiments were approved by the Ethics Committee for Scientific Research Involving Human Subjects and/or Animals, of the ”Lucian Blaga” University of Sibiu (process number 21/2024). The whole blood sample was previously centrifugated at 2500 rpm for 5 min to remove the plasma fraction and washed repeatedly with phosphate buffer (PBS) pH 7.4, until the supernatant became colorless. To the resuspended RBC in PBS buffer, aliquots of leaf extracts of different concentrations (serial dilutions of soft extract in PBS such as to obtain: 125, 250, 500 and 1000 μg/mL, respectively) were added. The samples were incubated for 2 h at 37 °C, then centrifuged at 2500 rpm for 5 min at r.t. A negative control sample (RBC in PBS) without extracts was used. Total lysis was obtained by incubation of RBC with distilled water (positive control). Absorbance of supernatants was measured at 576 nm using the spectrophotometer (Specord 200 Plus UV-Vis, Analytik Jena, Germany).

Percentage of hemolysis was calculated using the Equation (9):

(9) $$\%\;hemolysis=100\times \frac{A_{sample}-A_{control}}{A_{100}-A_{control}}$$

where

*A_sample_* = absorbance of the test sample (RBC in the presence of extract)
*A_control_*= absorbance of the control (RBC and PBS)
*A*_100_ = absorbance in case of total lysis (RBC and distilled water)

#### 3.4.2. In Vitro Biocompatibility and Cytotoxicity Assessment (MTS Assay)

The in vitro assessment of biocompatibility of extracts was performed on human gingival fibroblasts (HGF), while that of cytotoxicity was conducted on malignant melanoma (MeWo) and hepatocellular carcinoma (HepG2), all from CLS Cell Lines Service GmbH, Eppelheim, Germany, using the CellTiter 96^®^ AQueous One Solution Cell Proliferation Assay kit (Promega, Madison, WI USA). Cells were seeded in 96-well plates at 5 × 10^4^ cells/well (HGF) or 10^5^ cells/well (MeWo and HepG2) in alpha-MEM medium (PAN-Biotech GmbH, Aidenbach, Germany) supplemented with 10% fetal bovine serum (FBS) and 1% Penicillin-Streptomycin-Amphotericin B mixture (both from Gibco, Thermo Fisher Scientific, Waltham, MA, USA) and allowed to adhere for 24 h. Samples of *A. altissima* leaf extract were diluted in complete culture medium at 10, 50, 100, 250 and 500 µg extract/mL. Cells were then incubated for 24 h versus control cells (untreated). Control cells were incubated with a complete cell culture medium (considered 100% cell viability). An FLUOstar^®^ Omega microplate reader (BMG LABTECH, Ortenberg, Germany) was used to record the MTS absorbance at 490 nm 3 h after addition of the reagent. The viability of cells treated with different concentrations of the leaf extract was expressed as percentage (%) of the viability of control cells (means ± standard error of the mean). Experiments were done in triplicate.

#### 3.4.3. Membrane Leakage Assay from Lipid Vesicle

The membrane permeability properties were determined by measuring the induced leakage of 5(6)-carboxyfluorescein (CF) entrapped in small unilamellar vesicles (SUV) [121].

##### SUV Preparation

1,2-dioleyl-sn-glycero-3-phosphocholine (DOPC) was purchased from Avanti Polar Lipids, Inc. (Alabaster, AL, USA), while cholesterol (Ch) was a Sigma-Aldrich (St. Louis, MO, USA) product.

The lipid mixture DOPC/Ch (7/3) was dissolved in CHCl_3_ in a test tube, dried under N_2_, and lyophilized overnight. The lipid film was reconstituted with a solution of CF in 30 mM Hepes buffer (pH 7.4) at r.t. for 1 h. To make SUVs, the resulting multilamellar vesicle suspension was sonicated (GEX400 Ultrasonic Processor, Sigma) on ice until the initially cloudy lipid dispersion became translucent. The excess of fluorescent dye was eliminated by gel filtration on Sephadex G-75 (Sigma). SUVs were diluted to a concentration of 0.06 mM with Hepes buffer (5 mM Hepes, 100 mM NaCl, pH 7.4). The SUVs were stored at 4 °C and used within 24 h.

##### Leakage from SUV

The extract-induced leakage from SUVs was measured at 293 K using a CF-entrapped vesicle technique and a spectrofluorometer (Perkin Elmer model MPF-66). The phospholipid concentration was kept constant (0.06 mM) and increasing [extract]/[lipid] molar ratios were obtained by adding aliquots of extract solutions. The membrane leakage properties were examined for leaf extracts at 125, 250, 500 and 1000 μg/mL, respectively. The highest concentration (1000 μg/mL) was analyzed for two added volumes, 75 μL, investigated for all the concentrations, and 200 μL. After rapid and vigorous stirring, the time course of the fluorescence change corresponding to CF escape was recorded at 520 nm (6-nm band pass) with λ_exc_ 488 nm (3-nm band pass). The percentage of released CF at time *t* was determined according to formula (10):

(10) $$\%\;CF=100\times \frac{F_{t}-F_{0}}{F_{T}-F_{0}}$$

where

*F*_0_ = fluorescence intensity of vesicles in the absence of extract,
*F_t_* = fluorescence intensity of vesicles at time *t* in the presence of extract,
*F_T_* = total fluorescence intensity determined by disrupting the vesicles by addition of 50 µL of a Triton X-100 solution.

The kinetic experiments were stopped at 20 min.

### 3.5. Statistical Analysis

Experimental data with values expressed as mean ± SD were subjected to statistical analysis using the R 4.3.1 program [122], ANOVA, Kruskal–Wallis test, Tukey’s test, Dunn’s test for multiple comparisons/dunn.test package R Package Version 1.3.5. [123]. The correlation analysis was performed using the rcorr function of the Hmisc package R Package Version 5.1-0 [124]. The Spearman’s correlation was investigated for data which were not normally distributed. The function ggboxplot of the ggpubr package R Package Version 0.6.0 [125] was used to create representative boxplot graphs, while the correlogram was constructed using the corrplot.mixed function from the corrplot package Version 0.92 [126]. The LC_50_ values obtained from the brine shrimp (*A. salina*) assay were determined using the Probit analysis [127], in order to evaluate the toxicity of the leaf extracts. The results were considered statistically significant at *p* < 0.05.

## 4. Conclusions

The present study brings novelty and completes the existing information on potential toxicity of *A. altissima* leaves by using a complex approach towards different target/non-target organisms.

Extract of *A. altissima* leaves in ethanol solution showed a high content of compounds of polyphenolic structure, and antioxidant activity.

The extract exhibited low ecotoxic effects towards wheat, tomato, and parsley up to 500 μg/mL, and significant inhibitory effects on tomato germination at 1000 μg/mL. Screening *A. altissima* for ecotoxicity towards brine shrimp indicated a low toxicity at 24 h of exposure (Clarkson’s toxicity criterion based on LC_50_ values), which increased with exposure time and extract concentration. Cytotoxicity of the extract towards sheep erythrocytes indicate non-hematotoxicity. Moreover, the extract was found biocompatible with human gingival fibroblasts according to the MTS assay. Under the explored conditions, the leaf extract did not exhibit any capability to alter or disrupt liposomes within the tested concentration range, according to experiments on model lipid small unilamellar vesicles.

Toxicity tested against target organisms such as malignant melanoma and hepatocellular carcinoma indicates no anti-proliferative effect within the concentration range of 10 μg/mL to 500 μg/mL.

Given the low to moderate toxicity under the tested conditions, the autumn leaves of *A. altissina* could find useful practical applications/biopesticides. However, some limitations must be addressed, such as requirements for further studies on other cells than erythrocytes or gingival fibroblasts, and in vivo studies to confirm their safety for humans.

## Figures and Tables

**Table 1:** Retention time (RT) of polyphenolic compounds recorded in the individual polyphenol injections, in the polyphenol mixture, and in *A. altissima* leaf extract.

Polyphenols	RT (min)		
	Polyphenols as Individual Run	Polyphenols in Mixture Run	*A. altissima* Leaf Extract
Gallic acid	10.97	10.84	10.71
Protocatechuic acid	13.98	13.9	13.99
Catechin	15.63	15.91	15.79
Vanillic acid	16.74	overlapped with epicatechin	16.65
Epicatechin	17.28	17.38	-
Caffeic acid	17.49	17.96	-
Syringic acid	18.09	overlapped with caffeic acid	-
Rutin	20.42	20.63	20.7
Ferulic acid	22.38	22.35	-
*p*-Coumaric acid	23.1	23.46	23.48
Hesperidin	24.01	24.03	24.38
Rosmarinic acid	26.33	26.69	26.53
Salicylic acid	29.31	29.61	-
Quercetin	34.72	34.65	35.22
Kaempferol	39.71	41.0	-

**Table 2:** Wheat germination and growth parameters in the presence of *A. altissima* ethanolic leaf extract and in control sample.

Physiological Parameters *	Leaf Extract Concentration (μg/mL)				Control
	125	250	500	1000	
Eg (%)	62.00 ± 0.27	42.00 ± 0.19	40.00 ± 0.16	60.00 ± 0.18	48.00 ± 0.18
G (%)	86.00 ± 0.15	76.00 ± 0.13	68.00 ± 0.24	58.00 ± 0.17	72.00 ± 0.19
RL (cm)	7.89 ± 2.53	6.17 ±1.66	5.57 ± 1.65	5.10 ± 0.44	6.94 ± 1.46
SL (cm)	8.68 ± 1.76	7.68 ± 1.25	7.68 ± 2.39	8.91 ± 1.27	7.89 ± 1.65
RRG (%)	121.00 ± 0.54	91.00 ± 0.29	82.00 ± 0.25	71.00 ± 0.15	100.00 ± 0.00
RSG (%)	131.00 ± 0.57	121.00 ± 0.73	107.00 ± 0.62	72.00 ± 0.21	100.00 ± 0.00
GI (%)	108.03 ± 0.54	72.25 ± 0.39	57.48 ± 0.28	40.77 ± 0.13	72.00 ± 0.19
VI (%)	78.21 ± 0.23	61.59 ± 0.18	51.46 ± 0.27	33.14 ± 0.10	67.02 ± 0.27
PI (%)	152.00 ± 0.82	125.00 ± 0.87	113.00 ± 0.78	89.00 ± 0.45	100.00 ± 0.00

* Eg—Germinative energy; G—germination rate; RL—root length; SL—shoot length; RRG—relative root growth percentage; RSG—relative seed germination; GI—germination index; VI—vigor index; PI—influence index on the aerial part; Data are shown as the mean ± standard deviation.

**Table 3:** Tomato seeds germination and growth parameters in the presence of *A. altissima* ethanolic leaf extract and in control sample.

Physiological Parameters *	Leaf Extract Concentration (μg/mL)		Control
	125	1000	
Eg (%)	66.00 ± 0.05	60.00 ± 0.10	56.00 ± 0.05
G (%)	88.00 ± 0.13	84.00 ± 0.15	92.00 ± 0.08
RL (cm)	3.77 ± 1.05	1.12 ± 0.45	3.72 ± 0.69
SL (cm)	2.46 ± 0.47	2.14 ± 0.48	2.29 ± 0.43
RRG (%)	107.87 ± 0.45	32.12 ± 0.19	100.00 ± 0.00
RSG (%)	96.28 ± 0.17	91.56 ± 0.16	100.00 ± 0.00
GI (%)	96.25 ± 0.45	26.14 ± 0.14	92.00 ± 0.08
VI (%)	133.64 ± 0.27	44.01 ± 0.14	149.96 ± 0.15
PI (%)	111.64 ± 0.51	87.96 ± 0.34	100.00 ± 0.00

* Eg—Germinative energy; G—germination rate; RL—root length; SL—shoot length; RRG—relative root growth percentage; RSG—relative seed germination; GI—germination index; VI—vigor index; PI—influence index on the aerial part; Data are shown as the mean ± standard deviation.

**Table 4:** Parsley seeds germination and growth parameters in the presence of *A. altissima* ethanolic leaf extract and in the control sample.

Physiological Parameters *	Leaf Extract Concentration (μg/mL)		Control
	125	1000	
G (%)	52.50 ± 0.05	50.00 ± 0.17	56.00 ± 0.11
RL (cm)	1.05 ± 0.18	1.24 ± 0.66	1.47 ± 0.55
SL (cm)	0.77 ± 0.25	0.85 ± 0.65	0.90 ± 0.22
RRG (%)	71.52 ± 0.31	80.65 ± 0.44	100.00 ± 0.00
RSG (%)	88.01 ± 0.08	86.67 ± 0.23	100.00 ± 0.00
GI (%)	37.46 ± 0.16	43.19 ± 0.32	56.00 ± 0.11
VI (%)	73.94 ± 0.12	81.54 ± 0.44	92.64 ± 0.36
PI (%)	78.90 ± 0.43	84.36 ± 0.66	100.00 ± 0.00

* G—germination rate; RL—root length; SL—shoot length; RRG—relative root growth percentage; RSG—relative seed germination; GI—germination index; VI—vigor index; PI—influence index on the aerial part; Data are shown as the mean ± standard deviation.

**Table 5:** LC_50_ values of the brine shrimp exposed to different concentrations of *A. altissima* leaf extract, determined using the Log Concentration.

Concentration (μg/mL)	Concentration Logs (X)	Number of Organisms	Mortality Rate (%)		Probit (Y)		Probit (Y) Corrected *		LC_50_ (μg/mL)	
			24 h	48 h	24 h	48 h	24 h	48 h	24 h	48 h
250	2.40	10	0	17.00 ± 0.05	2.60	4.05	3.04 *	4.05	951.04 ± 28.26	420.65 ± 8.56
500	2.70	10	0	57.00 ± 0.05	2.60	5.18	3.04 *	5.18		
1000	3.00	10	37.00 ± 0.06	100.00 ± 0.00	4.67	7.40	4.67	6.96 *		
2000	3.30	10	100.00 ± 0.00	100.00 ± 0.00	7.40	7.40	6.96 *	6.96 *		

* corrected values of probit for mortality rates of 0% or 100% according to the method of Miller and Tainter (1944) [78] explained by Randhawa (2009) [79].

**Table 6:** Gradient elution of the polyphenols separation method using Agilent 1260 Infinity system.

% B	1	5	20	45	70	100	1	1
Time	0	2	10	40	55	75	80	90

## Data Availability

All of the data are contained within the article.
